# An ambient-temperature storage and stabilization device performs comparably to flash-frozen collection for stool metabolomics in infants

**DOI:** 10.1186/s12866-021-02104-6

**Published:** 2021-02-22

**Authors:** Sivapriya Ramamoorthy, Shira Levy, Masouma Mohamed, Alaa Abdelghani, Anne M. Evans, Luke A. D. Miller, Lopa Mehta, Sean Moore, Elizaveta Freinkman, Suchitra K. Hourigan

**Affiliations:** 1 Metabolon, 617 Davis Dr UNIT 100, Morrisville, NC 27560 USA; 2 Inova Children’s Hospital, 3300 Gallows Rd, Falls Church, VA 22042 USA; 3 Inova Health System, 3300 Gallows Rd, Falls Church, VA 22042 USA; 4 Division of Pediatric Gastroenterology, Hepatology, and Nutrition, Department of Pediatrics, University of Virginia, 200 Jeanette Lancaster Way, Charlottesville, VA 22903 USA; 5 Pediatric Specialists of Virginia, 3023 Hamaker Ct, Fairfax, VA 22031 USA

**Keywords:** Metabolomics, Stool, Microbiome, Infants, Ambient temperature

## Abstract

**Background:**

Stool metabolites provide essential insights into the function of the gut microbiome. The current gold standard for storage of stool samples for metabolomics is flash-freezing at − 80 °C which can be inconvenient and expensive. Ambient temperature storage of stool is more practical, however no available methodologies adequately preserve the metabolomic profile of stool. A novel sampling kit (OMNImet.GUT; DNA Genotek, Inc.) was introduced for ambient temperature storage and stabilization of feces for metabolomics; we aimed to test the performance of this kit vs. flash-freezing. To do this stool was collected from an infant’s diaper was divided into two aliquots: 1) flash-frozen and 2) stored in an OMNImet.GUT tube at ambient temperature for 3–4 days. Samples from the same infant were collected at 2 different time points to assess metabolite changes over time. Subsequently, all samples underwent metabolomic analysis by liquid chromatography – tandem mass spectrometry (LC-MS/MS).

**Results:**

Paired fecal samples (flash-frozen and ambient temperature) from 16 infants were collected at 2 time points (32 individual samples, 64 aliquots). Similar numbers of metabolites were detected in both the frozen and ambient temperature samples (1126 in frozen, 1107 in ambient temperature, 1064 shared between sample types). Metabolite abundances were strongly correlated between storage methods (median Spearman correlation Rs = 0.785 across metabolites). Hierarchical clustering analysis and principal component analysis showed that samples from the same individuals at a given time point clustered closely, regardless of the storage method. Repeat samples from the same individual were compared by paired t-test, separately for the frozen and OMNImet.GUT. The number of metabolites in each biochemical class that significantly changed (*p* < 0.05) at timepoint 2 relative to timepoint 1 was similar in flash-frozen versus ambient temperature storage. Changes in microbiota modified metabolites over time were also consistent across both methodologies.

**Conclusion:**

Ambient temperature storage and stabilization of stool in the OMNImet.GUT device yielded comparable metabolomic results to flash freezing in terms of 1) the identity and abundance of detected biochemicals 2) the distinct metabolomic profiles of subjects and 3) changes in metabolites over time that are plausibly microbiota-induced. This method potentially provides a more convenient, less expensive home collection and storage option for stool metabolomic analysis.

**Supplementary Information:**

The online version contains supplementary material available at 10.1186/s12866-021-02104-6.

## Background

Metabolites are critical signaling molecules that mediate host-microbiome communication and dynamics [1]. Bioactive metabolites produced by gut microbiota, such as short-chain fatty acids, aromatic amino acid metabolites, and bile acids, have been implicated in numerous human health conditions, including obesity [2], insulin resistance [3], cardiovascular disease [4], autism-spectrum disorder [5], and Parkinson’s disease [6]. Due to the heterogeneity of human genetics, microbiome composition, nutrient intake and other exposures, these studies have made only limited inroads into the richness and diversity of human gut metabolites. In addition to providing fundamental insights into disease pathology, these molecules may offer avenues to therapeutic strategies and biomarker discovery. Consequently, attention in the microbiome field is increasingly turning to metabolomics – the discipline of measuring metabolites in a comprehensive and non-targeted manner [7] – as a powerful tool to elucidate how the gut microbiome exerts its effects on human health and disease [8–10].

Despite this growing interest, the practical challenge of collecting fecal samples from human volunteers has constrained the application of metabolomics in gut microbiome research. In general, immediately freezing samples at − 80 °C has long been viewed as the optimal approach for preserving metabolites by quenching enzymatic activity, hydrolysis, oxidation, and other degradative processes [11]. Yet, in the case of human feces, obvious considerations of donor privacy and convenience have driven a strong demand for at-home collection. However, storage and shipping of frozen at-home collected samples can be inconvenient for participants and prohibitively expensive for researchers, pushing the need for ambient-temperature storage options. To fill this need, several research groups have recently sought to repurpose currently available sampling devices, such as DNA-stabilizing tubes and fecal immunochemical test (FIT) tubes [12, 13], for metabolomic analysis. However, the abundant detergents, buffers, salts, and other additives in these tubes render them incompatible with liquid chromatography and mass spectrometry (LC/MS), the technique of choice for metabolomic analysis. As a result, use of these devices significantly distorted the metabolomic profile of stool samples relative to the gold-standard flash-freezing methodology [12, 13].

Recently, OMNImet.GUT tubes (DNA Genotek, Inc.) were launched onto the market as a stool storage and stabilization kit developed specifically for metabolomics. We aimed to test the fidelity with which samples collected and stored in OMNImet.GUT tubes recapitulate the metabolomic profile of those collected by the gold-standard method, i.e., flash-freezing at − 80 °C. To accomplish this, we conducted a unique hospital-based field test in which matched samples (*n* = 16 donors, each donor sampled at two timepoints) were both flash-frozen and stored in OMNImet.GUT tubes. This study design enabled us to compare the two sample types directly (within-donor comparisons) as well as to compare groups of donors within each sample type (inter-donor comparisons) to assess whether the OMNImet.GUT samples would yield the same biochemical findings as the flash-frozen samples.

## Results

A total of 16 infants provided paired fecal samples (flash frozen and ambient) collected at 2 different time points (mean time between samples = 23 days, range 5–44 days), for a total of 32 individual samples and 64 aliquots. See Table 1 for demographic and clinical data for subjects.

**Table 1:** Characteristics of study cohort

Variable	Mean (range) or Frequency % ***n*** = 16
**Gender**	
Male	5/16 (31%)
Female	11/16 (69%)
**Ethnicity**	
Hispanic or Latino	3/16 (19%)
Not Hispanic or Latino	12/16 (75%)
Unknown	1/16 (6%)
**Race**	
White or Caucasian	6/16 (38%)
Black or African American	2/16 (12.5%)
Asian	1/16 (6%)
Other	5/16 (31%)
Declined to answer	2/16 (12.5%)
**Gestational age at birth (weeks)**	28 1/7 (24 5/7 35 4/7)
Extremely preterm (<27 6/7 weeks)	7/16
Early Pre-Term (28 0/7-33 6/7 weeks)	8/16
Late pre-Term (34 0/7 - 36 6/7 weeks)	1/16
Full Term (≥37 0/7 weeks)	0/16
**Delivery mode**	
Cesarean Section	13/16 (81%)
Vaginal Delivery	3/16 (19%)

**Fig. 1:**
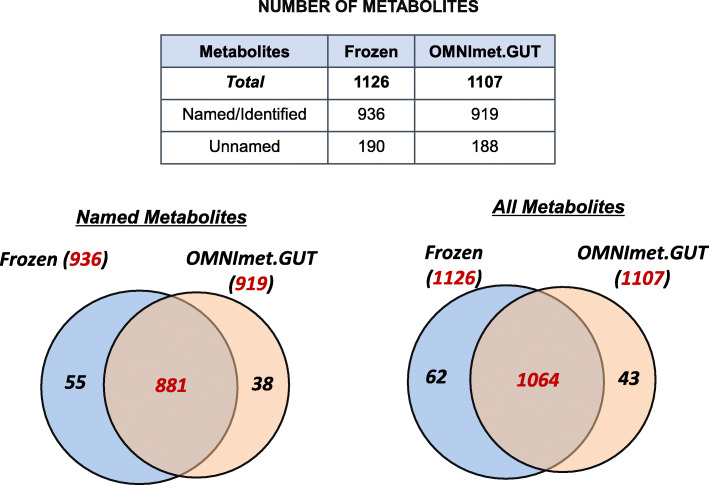
Venn Diagram Comparing all biochemicals detected in FrozPlease check if the Figure captions are presented correctlyen and OMNImet.GUT tube fecal samples

**Fig. 2:**
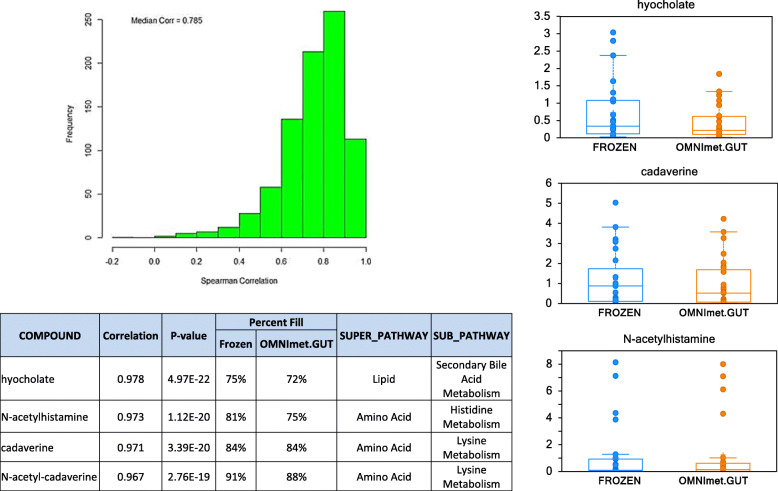
Correlation of metabolite abundance between in Frozen and OMNImet.GUT tube fecal samples. Hyocholate, N-acetylhistamine, cadaverine and N-acetyl-cadaverine were metabolites with high correlation values and were detected in more than 70% of the samples. A table including examples of the top metabolites that correlated between Frozen and OMNImet.GUT tube samples with their corresponding correlation value, *p*-value, percent fill, superpathway and subpathway are also shown. Representative plots for the top correlating metabolites show the level of the metabolite in Frozen and OMNImet.GUT tube samples

Similar numbers of metabolites were detected in both the frozen and ambient temperature samples, with a total of 1126 metabolites in frozen samples and 1107 in ambient temperature samples, of which 1064 were shared between the sample types, i.e. 94.5% of the metabolites detected in flash-frozen samples were also detected in the OMNImet.GUT samples (Fig. 1). These metabolites were classified into 10 super-pathways (all shared between both sample types) and 121 sub-pathways, of which 117 were shared between both sample types. Sixty-two metabolites from 7 super-pathways and 38 sub-pathways were only found in frozen samples; 43 metabolites from 8 super-pathways and 24 sub-pathways were only found in ambient temperature samples (Supplemental Table 1). Metabolite abundances were strongly correlated between storage methods, with a median Spearman correlation Rs = 0.785 across metabolites that were detected in at least 50% of samples of each type (Fig. 2, Supplemental Table 2).

**Fig. 3:**
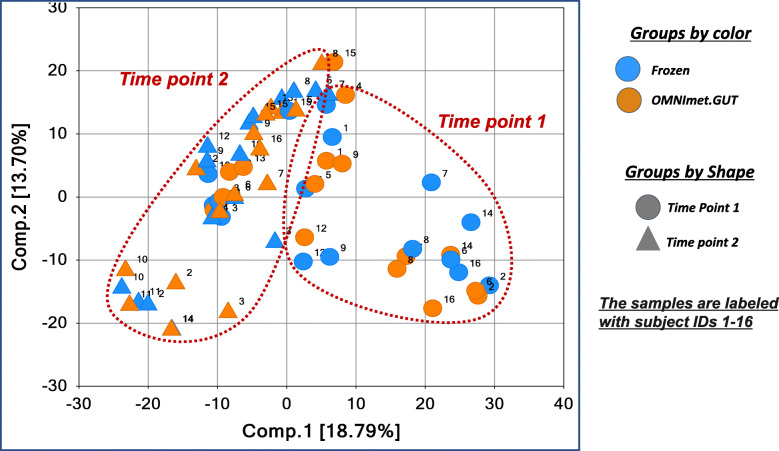
Principal Component Analysis (PCA) of Frozen vs. OMNImet.GUT fecal samples collected at 2 timepoints. The PCA was generated using the merged data including the 1064 metabolites detected in both the Frozen and OMNImet.GUT tubes

**Fig. 4:**
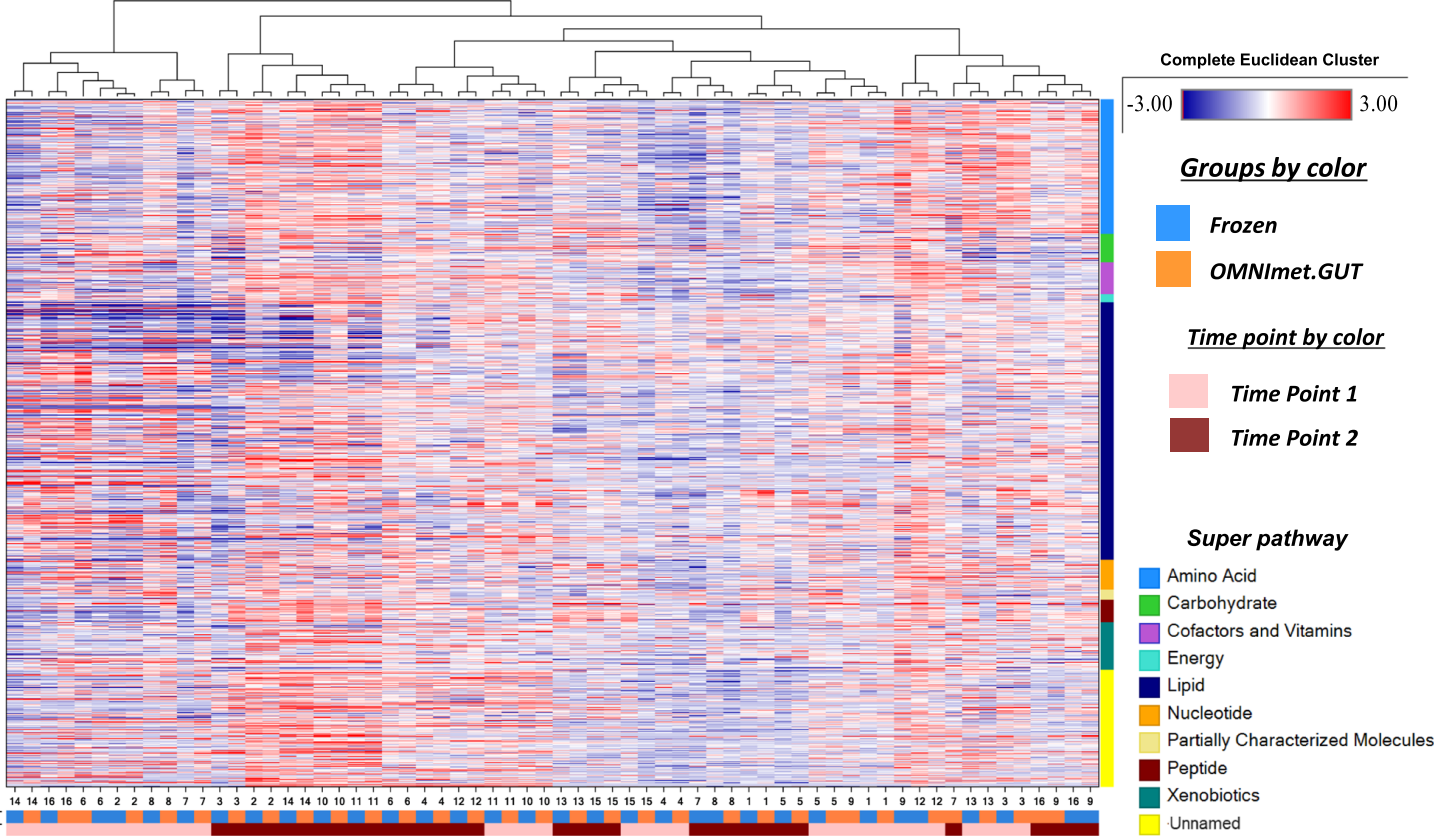
Hierarchical clustering analysis (HCA) of the relative abundance of metabolites in Frozen vs. OMNImet.GUT fecal samples collected at 2 timepoints. The HCA was generated using the merged data including the 1064 metabolites detected in both the Frozen and OMNImet.GUT tubes

**Fig. 5:**
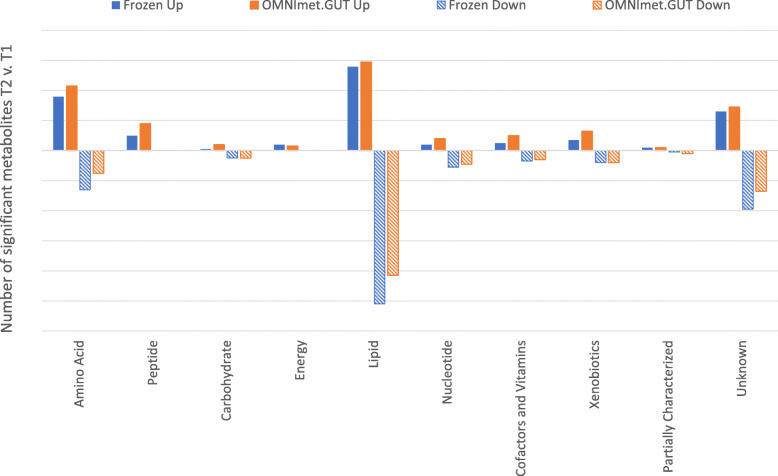
Repeat samples from the same individual were compared by paired t-test, separately for the Frozen and OMNImet.GUT collections. The number of metabolites in each biochemical class that increased (up; solid bars) or decreased (down; hatched bars) with *p* < 0.05 at timepoint 2 relative to timepoint 1 is plotted for Frozen (blue) and OMNImet.GUT (orange) sample sets

PCA and HCA of the 1064 metabolites detected in both frozen and OMNImet.GUT samples showed that the samples from the same individuals at a given time point clustered closely, regardless of the storage method (Fig. 3 and 4), suggesting that the effect of OMNImet.GUT storage on the individual metabolomic profiles was small compared to the differences among individual patients. Both HCA and PCA also showed a partial separation between samples from the two timepoints, also regardless of storage method, consistent with time-dependent metabolomic changes that occurred across multiple patients and were captured by both storage methods. To further investigate this time-dependent signature, repeat samples from the same individual were compared by paired t-test, separately for the frozen and OMNImet.GUT collections. The number of metabolites in each biochemical class that significantly changed (*p* < 0.05) at timepoint 2 relative to timepoint 1 was similar in flash frozen versus ambient temperature storage (Fig. 5).

**Fig. 6:**
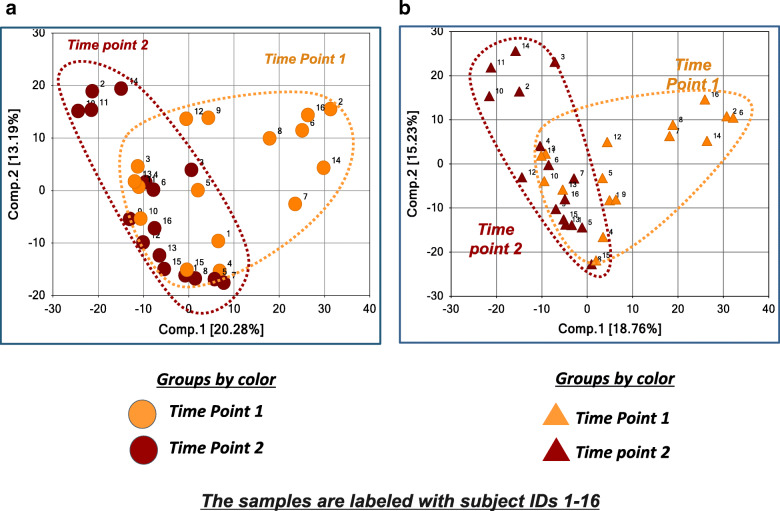
**a** Principal Component Analysis (PCA) of samples collected at 2 timepoints. 6A: The PCA was generated using the 1126 metabolites detected in the Frozen samples. **b** The PCA was generated using the 1107 metabolites detected in the OMNImet.GUT samples

To gain better insight into how fecal sample collection and storage for the frozen and OMNImet.GUT samples impacted biological changes related to timepoint 1 and timepoint 2 in the same individual, PCA analysis was performed separately for the frozen and OMNImet.GUT dataset. PCA showed major separation of a subset samples from the two time points on Component 1 for both frozen (Fig. 6a) and OMNImet.GUT samples (Fig. 6b), suggesting changes in metabolomic profiles between the time points mediated by developmental changes in the infants. Together, these observations suggest that time point might be a prominent contributor to variation in metabolomic profile in both the frozen and OMNImet.GUT tube samples**.** Importantly, regardless of storage method, the frozen samples and OMNImet.GUT tube-collected samples yielded strikingly similar PCA results, suggesting that the method of fecal storage did not greatly influence the biochemical signature.

**Fig. 7:**
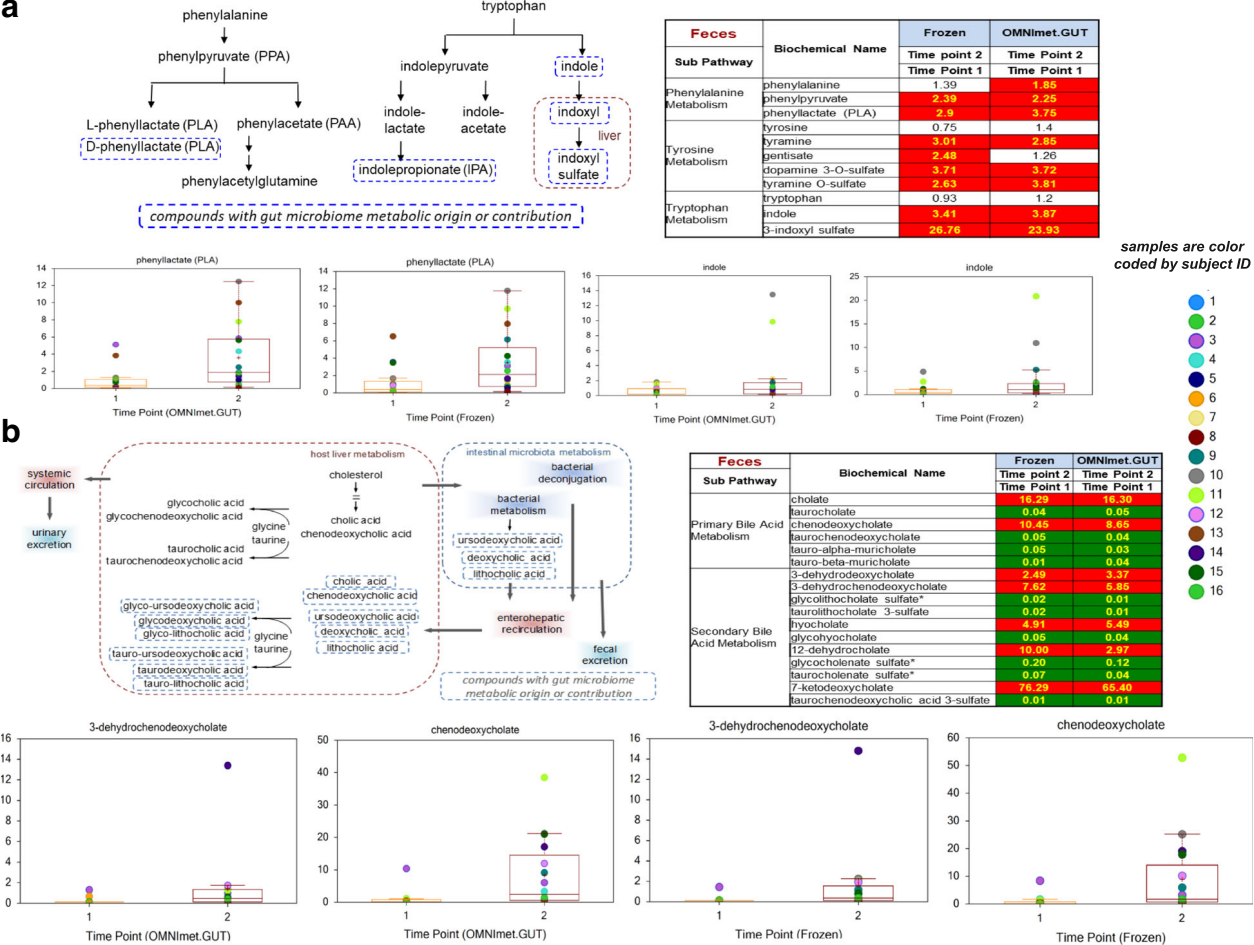
Changes in microbiome modified metabolites (**a**: aromatic amino acids, **b**: primary and secondary bile acids) between time points in both Frozen and OMNImet.GUT tubes samples. Heat map representation of statiscally significant metabolites between Time Point 1 and Time Point 2 in frozen and OMNImet.GUT tube samples. Significant (*p* ≤ 0.05) increases are indicated by red, while significant reductions are represented by green

With a clear distinction of metabolomic profiles between timepoint 1 and timepoint 2, we next determined if metabolites known to be modified by the gut microbiome were contributing to the time dependent changes. Changes in specific aromatic amino acids metabolites, which are derived from microbial metabolism of these amino acids, were observed between timepoint 1 and timepoint 2 in both frozen and OMNImet.GUT tube samples. Altered levels of phenyllactate (PLA), tyramine, indole and 3-indoxyl sulfate between timepoint 1 and timepoint 2 were seen (Fig. 7a). Furthermore, differences in primary and secondary bile acids (which are also modified by the gut microbiota) were seen between timepoint 1 and 2 in both storage methods (Fig. 7b). Overall, these results represent changes in metabolites between the two time points, captured by both storage methods that are plausibly microbiota-induced [14–16].

## Discussion

This is the first study to our knowledge to directly compare the fidelity of the new OMNImet.GUT device, designed for ambient temperature storage and stabilization of stool metabolites, with the current gold standard of flash freezing of stool. We found that metabolite analysis from the OMNImet.GUT device yielded comparable results to flash freezing in terms of 1) the identity and abundance of detected biochemicals, 2) the distinct metabolomic profiles of subjects, and 3) changes in metabolites over time that are plausibly microbiota-induced.

While the optimal method for metabolic profiling of stool is likely extraction within 1 h of collection [17], this method is out of reach in the vast majority of circumstances. It is therefore accepted that the next best method and more practical “gold standard” is flash-freezing of stool below − 20 °C [18]. However, flash-freezing of samples is inconvenient, expensive and not practical for large epidemiological studies. A handful of prior studies have attempted to address the feasibility of ambient temperature storage and storage for metabolite analysis using different storage methods. Loftfield et al. [13] performed metabolomic analysis of samples collected into 95% ethanol, fecal occult blood test cards (FOBT) and fecal immunochemical test tubes (FIT) relative to matched flash-frozen samples from 18 healthy adult volunteers. The 95% ethanol tubes performed the best, providing detection of 89% of the metabolites detected in flash-frozen samples, while collection into either FIT or FOBT led to a significant decline in both metabolite coverage and correlation to flash-frozen samples. Another study [12] of three healthy adults investigated whether a DNA-stabilizing device intended for ambient storage of stool for metagenomic analysis (OMNIgene.GUT) would also be suitable for metabolomics. These authors found a median Spearman correlation < 0.5 between flash-frozen samples and samples immediately frozen in the metagenomic analysis tubes (i.e., without room temperature storage), indicating that this storage method significantly distorted the metabolomic profile relative to flash-freezing. In addition to these low correlations, Lim et al. [12] also found that the differences among a single individual’s samples across storage methods were greater than those among the three individuals’ samples when the storage method was held constant, which precludes the use of these metagenomic tubes for discovery of metabolomic distinctions among individuals. Finally, Wang et al. [19] also compared 95% ethanol and OMNIgene.GUT with flash-freezing. Consistent with Lim et al., in this second study OMNIgene.GUT also performed poorly, providing detection of only 34.3% of the metabolites detected in flash-frozen samples and with a median interclass correlation coefficient (ICC) to flash-frozen of only 0.21. Surprisingly, however, in the study by Wang et al. [19], 95% ethanol also showed a median ICC of only 0.35, possibly due to practical difficulties inherent in using a “homemade” sampling methodology rather than a commercially available kit. Together, these studies demonstrated the lack of a commercially available, metabolomics-compatible methodology for ambient fecal collection and storage prior to the development of OMNImet.GUT storage device. In contrast, our study found that ambient temperature storage in the OMNImet.GUT device maintained both the identity and abundance of detected biochemicals (94.5% overlapping coverage, median Spearman R = 0.795) and the distinct metabolomic profiles of the subjects.

Moreover, we examined the changes in metabolites over time, including microbiome modified aromatic amino acid metabolites and bile acids [20, 21]. We found that sample collection and storage in the OMNImet.GUT device accurately retained the changes in gut-microbial metabolites such as phenyllactate (PLA) and indole over time compared to the gold standard methodology of flash freezing.

### Limitations

Although this field testing of the OMNImet.GUT device showed highly comparable results to the gold standard of flash freezing, this is not precisely the same as home collection of stool as trained lab staff conducted the aliquoting of samples. Therefore, further validation of the OMNImet.GUT device may be needed in samples collected at home in the by lay study participants. Additionally, it would be relevant in future studies to compare samples collected at home and stored in the OMNImet.GUT storage device versus samples collected and frozen at home in a domestic freezer and then shipped in a container to maintain the frozen state, until being stored at − 80 °C in a laboratory (as currently used in several studies for home collection). All subjects in this study were infants and the metabolite content of stool likely differs in older children and adults due to microbiome and diet differences. Further validation may be valuable in stool collected from adults.

## Conclusions

Ambient temperature storage and stabilization of stool in the OMNImet.GUT device yielded comparable metabolomic results to flash freezing within a neonatal population in terms of 1) the identity and abundance of detected biochemicals 2) the distinct metabolomic profiles of subjects and 3) changes in metabolites over time that are plausibly microbiota-induced. This potentially provides a more convenient and less expensive home collection and storage option for stool metabolite analysis, with many potential uses, including in longitudinal childhood microbiome research.

## Methods

As part of a longitudinal microbiome study conducted at the Inova Health System (IRB# 15–1945), infants who were admitted in the neonatal intensive care unit (NICU) were enrolled shortly after birth after written consent from their parent and had serial stool samples collected. Stool was collected from the infant’s diaper and divided into two aliquots, with an aim of approximately 500 mg per aliquot (range 120 mg–600 mg): 1) flash-frozen, i.e., immediately stored in an Eppendorf tube without preservatives at − 80 °C, and 2) stored in an OMNImet.GUT tube (DNA Genotek, Inc.) at ambient temperature for 3–4 days prior to freezing at − 80 °C. The time period of 3–4 days was chosen to simulate a situation in which samples are collected in a study subject’s home, which is the most convenient and user-acceptable location for fecal collection, and cannot be immediately frozen or delivered to a lab or clinic for freezing. A 3–4 day room-temperature storage window allows time for the study subject to hand-deliver or ship the sample to a study site. Samples from the same infant were collected at 2 different time points to assess changes in the metabolomic profile over time (mean time between samples = 23 days, range 5–44 days). OMNImet.GUT devices (product number ME-200) were obtained from DNA Genotek (Ottawa, ON).

To prepare feces for metabolomic analysis, frozen samples were lyophilized while OMNImet.GUT samples were dried in a Genevac evaporator. All dried samples were weighed and then resuspended at a 50:1 (50 μL deionized water for every 1 mg weight of feces) ratio for homogenization as previously described [13]. The homogenates were subjected to automated biochemical extraction and analysis by liquid chromatography and high-resolution tandem mass spectrometry (LC-MS/MS) on Metabolon’s Global Platform, as previously described [22–24]. Using proprietary software, raw data were extracted, peak-identified, and processed by Metabolon as previously described [13, 25–27]. Briefly, identification of metabolites was made by comparison to library entries of purified standards or recurrent unknown entities. A proprietary reference library of > 4500 known metabolites and > 2000 novel metabolites is maintained by Metabolon. Library entries contain the retention index (RI), mass to charge ratio (m/z), and spectral data (including MS/MS fragmentation). Three criteria were used for biochemical identification as follows: retention index within a narrow retention window, accurate mass (m/z; ±10 ppm), and the matching MS/MS spectra between the authentic standards and the experimental data. Comparison of the ions present in the experimental spectrum to the ions present in the library spectrum gave the MS/MS scores. Three types of controls were included: a pool of small portions of each experimental sample used as a technical replicate throughout the platform run; process blanks of extracted water samples; and a set of deuterium labeled standards spiked into every sample included in the project allowing instrument performance monitoring.

After identification, metabolites were quantified by peak integration. To analyze the Frozen and OMNImet.GUT sample sets separately, the data were scaled to a median of 1 for each biochemical in that sample set. Missing values, if any, were imputed with the observed minimum for that particular metabolite in that sample set. The data were natural log-transformed prior to statistical analyses including paired t-test and principal component analysis (PCA). To directly combine the Frozen and OMNImet.GUT sample sets, each set was normalized to a bridging control samples, which was prepared by pooling the extracts from the Frozen samples and was analyzed in *n* = 4 technical replicates in parallel with both of the experimental sets. In each set, metabolites were retained if present in at least 3 of the 4 bridging samples; for each metabolite, its raw peak areas were divided by the median of the raw peak areas across the bridging samples. After combining the two sample sets, only metabolites present in both sets were retained; for each metabolite, its missing values were imputed with its minimum value. The resulting merged dataset was natural log-transformed prior to statistical analyses including paired t-test, PCA, hierarchical clustering analysis (HCA) using complete clustering with Euclidean distance and Spearman correlation.

The datasets generated and/or analyzed during the current study are available in the Metabolights repository, (https://www.ebi.ac.uk/metabolights/MTBLS201).

## Supplementary Information

**Additional file 1: Supplemental Table 1.** Metabolites only detected in one collection method.

**Additional file 2: Supplemental Table 2.** Correlation of metabolite abundance between in Frozen and OMNImet.GUT tube fecal samples for individual metabolites.

## Data Availability

The datasets generated and/or analyzed during the current study are available in the Metabolights repository, (https://www.ebi.ac.uk/metabolights/MTBLS201).

## Abbreviations

PCA: Principal Component Analysis
HCA: Hierarchical Clustering Analysis
